# Remote Ischemic Preconditioning Protects Cisplatin-Induced Acute Kidney Injury through the PTEN/AKT Signaling Pathway

**DOI:** 10.1155/2019/7629396

**Published:** 2019-11-03

**Authors:** Wanfen Zhang, Cheng Chen, Ran Jing, Tongqiang Liu, Bicheng Liu

**Affiliations:** ^1^Division of Nephrology, The Affiliated Changzhou No. 2 People's Hospital of Nanjing Medical University, Changzhou, Jiangsu, China; ^2^Institute of Nephrology, Zhong Da Hospital, Southeast University, School of Medicine, Nanjing, Jiangsu, China

## Abstract

Although cisplatin (Cis) is an effective chemotherapeutic agent in treatment of various cancers, its adverse effect of nephrotoxicity limits the clinical application. Remote ischemic preconditioning (RIPC) is a strategy to induce resistance in a target organ against the oxidative stress and injury by applying transient, brief episodes of ischemia. However, whether RIPC exerts protective effect on Cis-induced renal injury remains unclear. In this study, we showed that RIPC significantly alleviated the renal functional and histopathological damage of Cis-induced acute kidney injury (AKI) mice. Furthermore, RIPC substantially reversed the downregulation of miR-144 and upregulation of PTEN in renal tissues of Cis-induced AKI mice and alleviated tubular cell apoptosis via activating PTEN/AKT signaling. In mechanism, we demonstrated that miR-144 directly targets the 3′-UTR of PTEN mRNA, and then the elevation of miR-144 in RIPC activates PTEN/AKT signaling by downregulating PTEN expression to achieve its antiapoptosis effect. Collectively, our results indicate that RIPC may be a potential therapeutic strategy in Cis-induced AKI, and provide insights on the underlying molecular mechanisms of cisplatin's nephrotoxicity.

## 1. Introduction

Cis-diamminedichloroplatinum(II) (cisplatin, Cis) is a classic chemotherapeutic agent with a widely clinical application in various tumors including ovarian, head and neck, testicular, and uterine cervical carcinomas [1]. However, its effects such as causing nausea and vomiting, and tissue and organ toxicity, limit the clinical application of cisplatin regardless of its potential medicinal effects [2]. Previously, approximately 20–30% of the patients who received a cisplatin administration exhibited acute kidney injury (AKI) [3, 4], which has become the most intractable problem in the application of cisplatin. Recently, independent groups found that many traditional Chinese medicines or their major compounds show well protective effects on Cis-induced kidney injury *in vivo*, such as QiShenYiQi, red ginseng, celastrol, and hesperetin [5–11]. Even though different strategies to prevent nephrotoxicity of cisplatin have been being developed, the hydration and forced diuresis remain to be the most commonly used ones. Therefore, how to prevent Cis-induced AKI without reducing its antitumor effects shows crucial practical theoretical significances.

Remote ischemic preconditioning (RIPC) is a strategy of applying transient, brief episodes of ischemia to induce resistance in a target organ against the oxidative stress and injury caused by a larger ischemic insult [12]. It has been showed that RIPC exerts protective effects on ischemia/reperfusion (IR) or contrast agent-induced AKI in rodents [13, 14]. Recently, several meta-analysis reviews with over 6000 patients demonstrated that RIPC may decrease the occurrence of AKI in cardiovascular surgery patients [15, 16]. In mechanism, Cai et al. have reported that RIPC induces late protection against myocardial IR injury by inactivating PTEN, a crucial negative upstream regulator of PI3K/AKT signaling by antagonizing the activity of PI3K. PI3K, as an intracellular phosphatidylinositol kinase, regulates cell growth, proliferation, differentiation, motility, and survival [17]. AKT, also known as protein kinase PKB, is an important downstream target of the PI3K signaling pathway. In fact, Gao et al. have demonstrated that PI3K/AKT signaling also plays an important role in the pathogenesis of kidney injury by regulating tubular cell apoptosis and inflammation [5]. Besides, merging evidences have showed that pharmacological inhibition of PTEN aggravates acute kidney injury both in IR-induced and Cis-induced AKI [18–20]. These findings imply that RIPC may exhibit protective effects on Cis-induced AKI animal model. If so, how RIPC influence PTEN/AKT activity needs to be determined.

MicroRNAs (miRNAs), a small noncoding RNA molecule (consisting of around 22 nucleotides), functions as a crucial posttranscriptionally regulatory mechanism by degrading the mRNA or inhibiting translation in most organisms [21, 22]. Early in 2013, miR-144 has been observed promoting cell proliferation, migration, and invasion in nasopharyngeal carcinoma by directly targeting the mRNA of PTEN [23]. MiR-144 also contributes to the pathogenesis of insulinomas via degrading PTEN mRNA to activate PTEN/AKT signaling [24]. More interesting, Li et al. observed that the expression of miR-144 was upregulated after RIPC, and the elevated miR-144 undertook cardioprotective functions by increasing PTEN/AKT signaling activity [25]. Therefore, we supposed that miR-144 may mediate the protective effects of RIPC on Cis-induced AKI in a PTEN/AKT pathway-dependent manner.

In this study, we first evaluated the protective effect of RIPC on Cis-induced AKI mouse models and found RIPC significantly alleviated the injury induced by cisplatin. Also, we observed that the expression of miR-144 and PTEN was increased and decreased, respectively, after RIPC surgery. Finally, our results highlighted a molecular mechanism that miR-144 directly targets the mRNA of PTEN and then activates the PTEN/AKT pathway, by which RIPC protects animals from Cis-induced injury. Collectively, RIPC may be a novel potential strategy for protecting Cis-induced kidney injury.

## 2. Materials and Methods

### 2.1. Animals

Male C57BL/6 mice, 8-12 weeks of age, were obtained from the Animal Center of Fudan University, Shanghai, China. Animals were housed in acrylic cages with shredded corn cob bedding in an acclimatized room (12/12 h light/dark cycle; 25 ± 1°C), provided with plenty water and fed with mouse breeder chow ad libitum, according to standard protocols for animal care. All the animal experimental protocols were in compliance with the Guide for the Care and Use of Laboratory Animals published by the Ethics Committee of Southeast University.

### 2.2. Grouping and Experimental Protocol

**Figure 1:**
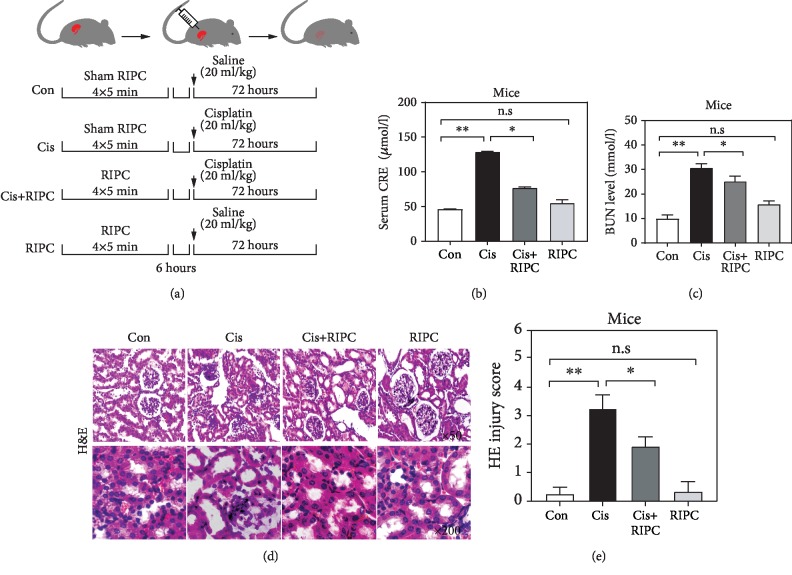
RIPC alleviates the Cis-induced AKI in mice. (a) The schematic representation of the experimental procedure involved in the study. (b, c) RIPC significantly reduced the increased CRE level (a) and BUN level (b) induced by cisplatin. *n* = 6; ^∗^*P* < 0.05, ^∗∗^*P* < 0.01; n.s: no significance in statistic. (d, e) RIPC alleviated Cis-induced kidney injury. Representative photomicrographs of tubular cell injury in mouse kidney tissue sections with H&E staining (d) and their quantification results (e). Original magnification: ×50 (top), ×200 (bottom). *n* = 6; ^∗^*P* < 0.05, ^∗∗^*P* < 0.01; n.s: no significance in statistic.

The RIPC surgery was operated in compliance with the previous literature [24]. Briefly, the skin was longitudinally incised on the right femoral triangle under anesthesia using microvascular clamping of the femoral vascular bundle, which consists of four cycles of 5 min ischemia followed by 5 min reperfusion. The sham operation for RIPC included all surgical procedures or treatments except for the clamping of the femoral arteries. Mice were randomly divided into four groups as follows:(1) Con, mice were subjected to the sham operation for RIPC before saline administration (20 ml/kg); (2) Cis, mice were subjected to sham operation for RIPC before Cis administration (20 mg/kg); (3) Cis+RIPC, 6 h after RIPC, mice were subjected to Cis administration (20 mg/kg); and (4) RIPC, mice were subjected to RIPC before saline administration (20 ml/kg). This procedure was visualized in Figure 1. 72 h after Cis administration, the animals were sacrificed.

### 2.3. Renal Function Measurement

In the present study, serum samples were used for the measurement of renal function including two variables: serum CRE and BUN. The blood samples (about 1.0 ml) were collected from the abdominal aorta or the jugular vein of the experimental animals. After a 30 min of clotting, the serum was obtained by centrifuging the samples at 2000 g for 10 min. Serum CRE was measured using a commercial creatinine assay kit (BioAssay Systems, Hayward, CA), and BUN was determined fluorometrically as the previously described (Yu et al., 2015) [26]. Briefly, equal volume (25 *μ*l) of ddH_2_O, serum sample, and standard application solution were added to the wells of 96-well plate, and 200 *μ*l of enzyme solution buffer was added to each well, and then mixed and incubated at 37°C for 10 min. Subsequently, the phenol developer (1 ml) and basic sodium hypochlorite (1 ml) were sequentially added to each well, and then mixed and incubated at 37°C for 10 min. Finally, the concentration of each well was determined using a microplate reader at a wavelength of 546 nm. As for BUN, equal volume (20 *μ*l) of ddH_2_O, serum sample, and standard application solution were added to the wells of 96-well plate. Then, enzyme solution A (150 *μ*l) was added to each well, mixed, and incubated at 37°C for 5 min. The absorbance value A1 was determined using a microplate reader at a wavelength of 546 nm. After adding the enzyme solution B (50 *μ*l) and incubating for 5 min at 37°C, the samples were analyzed using a microplate reader at 546 nm.

### 2.4. Histopathological Analysis

The kidneys were removed and fixed in 4% paraformaldehyde, embedded in paraffin, and sliced into 4 *μ*m sections. Sections were stained with hematoxylin and eosin (H&E) for morphologic analysis. A pathologist blinded to the study protocol analyzed the sections using a light microscope (Leica DM 6000 B; Leica Microsystems, Germany). For semiquantitative analysis of morphological changes, we randomly selected 10 high-magnification (×50, ×200) fields of the cortex and the outer stripe of the outer medulla. Tubule injury scores were referred to the previous report based on the percentage of tubules affected as follows: 0 ≤ 10%, 1 = 10‐25%, 2 = 26‐50%, 3 = 51‐75%, and 4 ≥ 75% [27].

### 2.5. Real-Time Quantitative PCR (qPCR)

The total RNA, containing miRNA, was extracted from kidney tissues or cells using TRIzol reagent (Takara, Japan) according to the manufacturer's instruction. The reverse transcription was performed using a PrimeScript RT Reagent kit (Takara, Japan). Real-time qPCR was performed on a Bio-Rad real-time PCR machine using IQ SYBR green supermix reagent (Bio-Rad, Hercules, CA). The expression levels of miRNAs and mRNAs were normalized to GAPDH level in each sample. All the qPCR reactions were run in triplicate.

### 2.6. Immunohistochemical Staining

Immunohistochemical staining was performed on paraffin sections. Antigen retrieval was performed with antigen unmasking solution (Vector Laboratories). Endogenous peroxidase activity was quenched with 3% H_2_O_2_. After blocked with 5% normal serum, the slides were incubated with primary antibodies in a humidified chamber overnight. The next day, the slides were incubated with appropriate secondary antibodies and ABC solution using ABC Elite kit (Vector Laboratories). The immunoreactivity was revealed by incubating the slides in DAB solution. Nuclear staining was performed using hematoxylin. The slides were dehydrated, cleared, and mounted. The images were acquired and analyzed by NIS Element software with Nikon microscope image system (Nikon Instruments).

### 2.7. Western Blotting

The protein extraction and western blotting were performed as the previous description [13]. The primary antibodies anti-PTEN, anti-p-PTEN, anti-p-AKT, anti-AKT, anti-p-GSK3*β*, anti-GSK3*β*, anti-BAX, and anti-BCL-2 were purchased from Cell Signaling Technology. Anti-GAPDH was purchased from KangChen Bio-tech, and anti-p-PI3K was purchased from Abcam. The dilution was in compliance with the manufacturer's instruction.

### 2.8. Immunofluorescence

After fixation in acetone for 10 min, the sections were treated with normal donkey serum for 50 min. Subsequently, the sections were incubated with primary antibody (p-GSK3*β*, PTEN, and cleaved caspace-3, respectively) at 4°C overnight. The next day, the sections were then incubated with secondary antibodies conjugated to Alexa Fluor 488 or Alexa Fluor 568 (1 : 200; Abcam), followed by nuclei counterstaining with 4′,6-diamidino-2-phenylindole (DAPI, 2 mg/ml). The slides were then examined using a fluorescence microscope (Leica TCS SP5).

### 2.9. Apoptosis Detection

Cell apoptosis was determined using terminal deoxynucleotidyl transferase-mediated dUTP nick-end labeling (TUNEL) staining, preformed on a DeadEnd Colorimetric Apoptosis Detection System (Millipore, Billerica, MA, USA) according to the manufacturer's instruction. The number of TUNEL-positive cells per high-power field was counted and analyzed in a blinded fashion.

### 2.10. Cell Culture and Luciferase Assays

The renal tubular epithelial cell NRK52 was purchased from ATCC. The NRK52 cells were maintained in Dulbecco's modified Eagle's medium (DMEM) containing 10% fetal bovine serum (FBS) and 1% penicillin/streptomycin in an incubator with 5% CO_2_ at 37°C. The medium was replaced when cells adhered to the bottle wall. The cells were subcultured until the cells covered 80% of the bottle bottom. For the transfection, NRK52 cells were transfected with synthetic miRNA miR-144 and the negative control (NC) (Ambion Pre-miR miRNA precursors; Life Technologies) using Lipofectamine 2000 (Invitrogen).

To generate the wild-type and mutant PTEN luciferase reporter constructs, the 3′-UTRs were cloned and inserted into pRL-NULL vectors with the appropriate restriction enzymes. The cells were seeded on 24-well plates at 5 × 10^5^ cells per well and cotransfected with 10 ng pRL-NULL-3′-UTR (untranslated region) plasmids and 100 ng of pGL3-control vector. The cells were harvested at 24 hours after transfection, and luciferase activities were measured. The values were normalized using the dual-luciferase reporter assay system according to the manufacturer's protocol (Promega).

### 2.11. In Vivo Inhibition of miRNA

Single-stranded RNA oligos were synthesized by VBC Biotech (Vienna), including antago-miR-144 (5′-agU ACA UCA UCU AUA Cug ua-Chol-3′) and a scrambled (mutated) miRNA as a control (antago-miR-Co/miR-Co: 5′-aaG GCA AGC UGA CCC UGA aguu-Chol-3′). Each oligonucleotide was deprotected, desalted, and purified by high-performance liquid chromatography. The oligos were injected via the tail vein at a dose of 20 mg/kg in 0.2 ml saline 2 h before RIPC.

### 2.12. Statistical Analysis

All data were expressed as mean ± SEM. Multiple group comparisons were performed by ANOVA followed by the Bonferroni procedure for comparison of means. Comparisons between two groups were analyzed by Student's *t*-test. *P* < 0.05 was considered as statistical significance.

## 3. Results

### 3.1. RIPC Alleviates the Cis-Induced AKI in Mice

To investigate the protective effects of RIPC on Cis-induced AKI mice, we constructed a RIPC pretreatment animal model following the procedure in Figure 1(a). It was frequently observed that the Cis-induced AKI mice exhibit the elevation of serum creatinine (CRE) and blood urea nitrogen (BUN) level, which are closely linked to renal function [28, 29]. Indeed, we observed that the serum CRE and BUN concentrations were significantly increased to 2.9- and 4.3-fold of Con group after Cis treatment (20 mg/kg) (Figures 1(b) and 1(c)), respectively, suggesting the kidney and animals receive a serious damage. Intriguingly, the deleterious effects of Cis were obviously reversed by RIPC treatment, indicated by the reduction of serum CRE and BUN levels compared with the Cis group (Figures 1(b) and 1(c)). Moreover, RIPC isolation did not influence the serum CRE and BUN levels (Figures 1(b) and 1(c)). We also checked the protective effects of RIPC on the renal structure of Cis-induced AKI mice using H&E staining. We found that Cis treatment resulted in the severe detachment and foamy degeneration of tubular cells in the renal cortex and the outer stripe of the outer medulla (Figures 1(d) and 1(e)). Interestingly, RIPC treatment obviously alleviated the severity of renal structural damage, indicated by the better tubular integrity compared with that of the Cis group (Figures 1(d) and 1(e)). We also observed that the group of RIPC isolation exhibited a similar renal structural integrity with the Con group (Figures 1(d) and 1(e)), suggesting it has no obvious nephrotoxicity. Altogether, our findings demonstrate that RIPC attenuates the Cis-induced functional and structural injury of kidney in mice.

### 3.2. RIPC Elevates miR-144 but Attenuates PTEN Expression in the Renal Tissues of Cis-Induced AKI Mice

**Figure 2:**
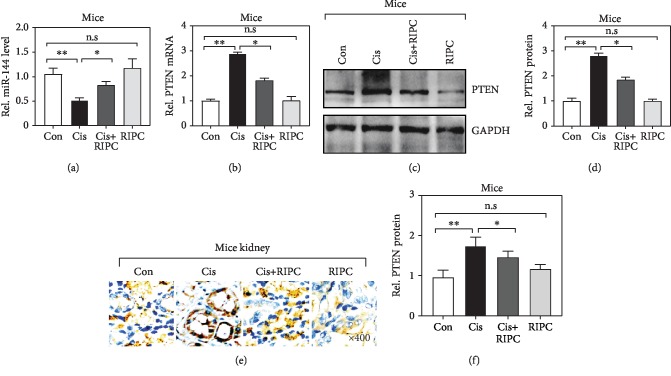
RIPC elevates miR-144 but attenuates PTEN expression in the renal tissues of Cis-induced AKI mice. (a, b) miR-144 and PTEN level was determined using real-time qPCR. *n* = 6; ^∗^*P* < 0.05, ^∗∗^*P* < 0.01; n.s: no significance in statistic. (c) The protein level of PTEN was determined by western blot. (d) Quantitative analysis of PTEN protein levels in (c). *n* = 6; ^∗^*P* < 0.05, ^∗∗^*P* < 0.01; n.s: no significance in statistic. (e) Representative photomicrographs of kidney sections stained with PTEN (brown) and counterstained with hematoxylin (blue) (original magnification, ×400). (f) Quantitative analysis of PTEN protein levels in (e). *n* = 6; ^∗^*P* < 0.05, ^∗∗^*P* < 0.01; n.s: no significance in statistic.

To explore the molecular mechanism by which RIPC achieves its protective biofunction for the kidney, we first determined the expression of miR-144 and PTEN using real-time qPCR. We observed that miR-144 was significantly downregulated but PTEN upregulated after Cis administration (Figures 2(a) and 2(b)), which is consistent to previous reports in other renal injury model. Intriguingly, the reduced miR-144 and elevated PTEN mRNA level were significantly reversed after RIPC treatment (Figures 2(a) and 2(b)). Also, we checked the protein level of PTEN using western blotting (WB) (Figures 2(c) and 2(d)). Consistently, we also observed the protein level of PTEN was obviously increased in the renal tissues from Cis-administrated mice (Figures 2(c) and 2(d)). This finding was further validated by PTEN immunostaining, especially in the tubular cells (Figures 2(e) and 2(f)). Altogether, these observations suggest that the aberrant expression of miR-144 and PTEN induced by administration was alleviated by RIPC treatment, implying they may involve in the pathogenesis of Cis-induced AKI.

### 3.3. MiR-144 Activates AKT/GSK3*β* Pathway by Directly Targeting PTEN

**Figure 3:**
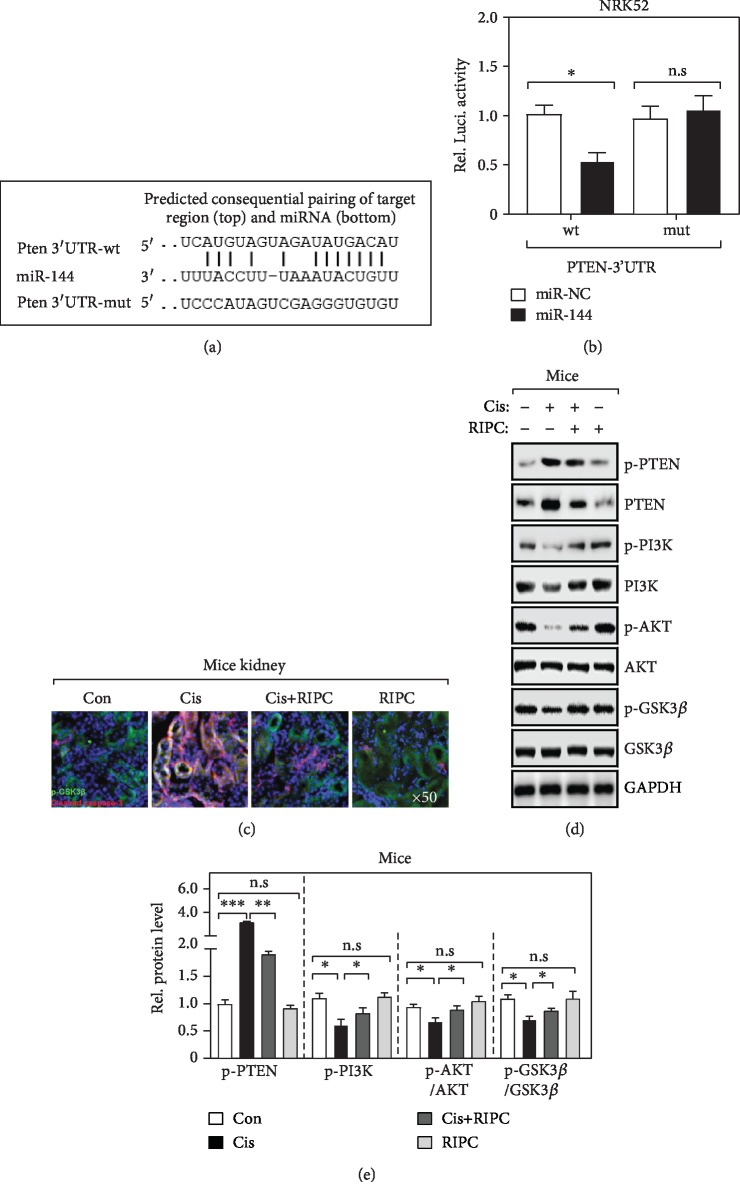
MiR-144 activates the AKT/GSK3*β* pathway by directly targeting PTEN (a) The sequence alignment of miR-144 and the 3′-UTR of PTEN mRNA. Wild-type (wt) and mutant (mut) sequences were used in (b). (b) Luciferase activities determined the binding between miR-144 and 3′-UTR of PTEN. NRK52 cells were cotransfected with the indicated plasmids. Each firefly luciferase activity was normalized by the cotransfected Renilla luciferase activity. *n* = 6; ^∗^*P* < 0.05; n.s: no significance in statistic. (c) Representative confocal image of immunofluorescence staining with p-GSK3*β* and cleaved caspase-3 in kidney sections. (d, e) Western blot determined the indicated protein level (d) and their quantification (e). *n* = 6; ^∗^*P* < 0.05; ^∗∗^*P* < 0.01; ^∗∗∗^*P* < 0.001; n.s: no significance in statistic.

To further validate that miR-144 and PTEN play roles in the pathogenesis of Cis-induced AKI, we first conducted a bioinformatics analysis of miR-144's targets using three independent algorithms. Interestingly, we found that miR-144 directly targets the 3′ untranslated region (3′-UTR) of PTEN's mRNA (Figure 3(a)). Consistently, our dual-luciferase assay also experimentally confirmed this finding. As shown in Figure 3(b), only the luciferase generated by plasmid ligated with the wild-type but not miR-144 binding site mutated 3′-UTR was specifically downregulated by the ectopic miR-144 in NRK52E cells, widely used renal tubular epithelial cells. Recently, it was frequently reported that the PTEN/AKT pathway is inactivated in the apoptotic renal tubular epithelial cells in AKI animal model [16, 20]. Also, we found that, after Cis treatment, the cleavage caspase-3 was significantly elevated determined by immunofluorescence (IF) analysis (Figure 3(c)), suggesting that the nephrocyte apoptosis is enhanced. Besides, our results showed that the PTEN/AKT pathway was activated after Cis administration, indicated by the elevation of PTEN, p-GSK3*β*, and p-AKT determined by WB analysis (Figures 3(d) and 3(e)). Intriguingly, these effects were substantially inhibited by RIPC treatment (Figures 3(d) and 3(e)). Altogether, our results demonstrate that miR-144 interferes PTEN's expression and then modules PTEN/AKT activity.

### 3.4. RIPC Protects Cis-Induced AKI Animals via Elevating miR-144 Expression

**Figure 4:**
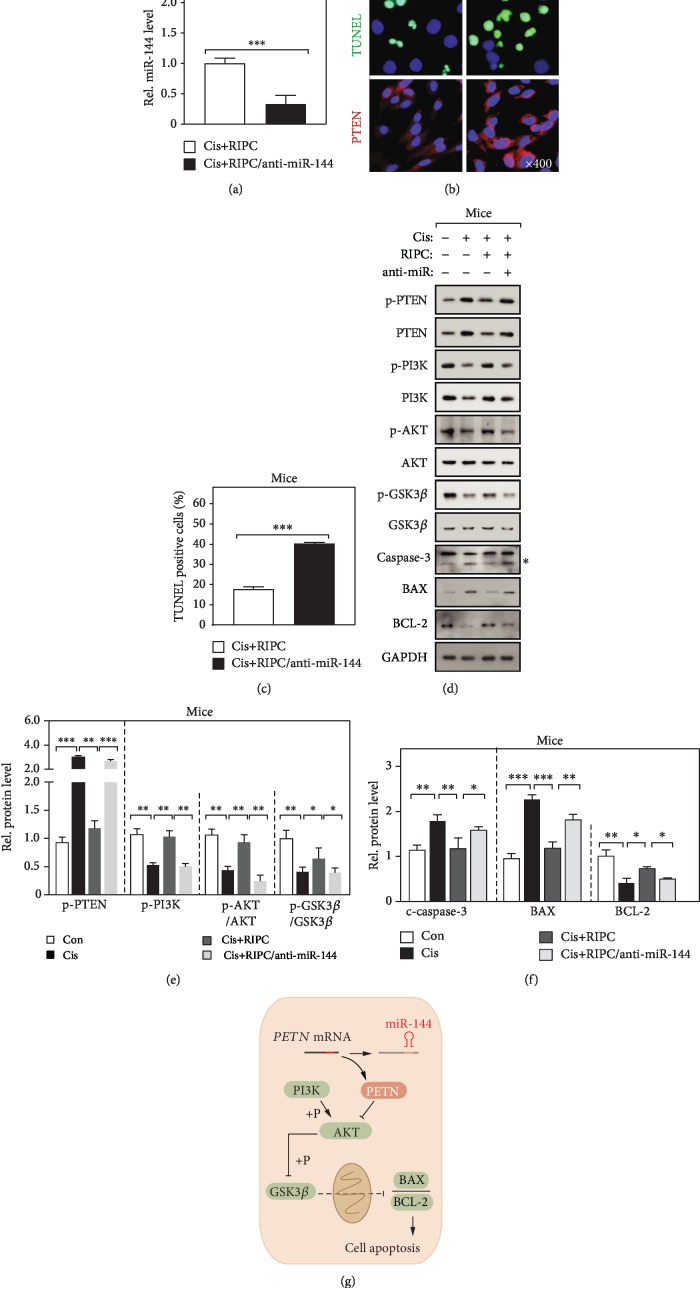
RIPC protects Cis-induced apoptosis via elevating miR-144 expression (a) Real-time PCR determined the expression of miR-144 in mouse kidney. *n* = 6; ^∗∗∗^*P* < 0.001. (b, c) Representative confocal image of TUNEL and PTEN immunofluorescence staining (b) and the quantitation analysis of TUNEL-positive cells (c). *n* = 6; ^∗∗∗^*P* < 0.001. (d, e) Western blot determines the indicated protein level (d) and the quantification of protein band (e). *n* = 6; ^∗^*P* < 0.05; ^∗∗^*P* < 0.01; ^∗∗∗^*P* < 0.001. The asterisk indicates the cleaved caspase-3. (g) The schematic representative of work model.

It was well documented that tubular epithelial cell apoptosis plays crucial roles in the pathogenesis of Cis-induced AKI [30, 31]. To further demonstrate that RIPC protects the kidney from Cis-induced AKI in a miR-144-mediated manner, we manipulated the expression of miR-144 using a well-worked antago-miR [28, 29]. Firstly, we checked the expression of miR-144 using real-time qPCR and found its expression was dramatically downregulated (Figure 4(a)). Also, we observed that loss function of miR-144 obviously blocks the protection effect of RIPC treatment on Cis-induced AKI animals, determined by the enhancement of tubular epithelial cell apoptosis and PTEN protein level of evaluated by IF staining (Figures 4(b) and 4(c)). Furthermore, we determined the activity of the PTEN/AKT signaling pathway and found significantly increasing in the miR-144 loss function group (Figures 4(d) and 4(e)). Taken together, our observations indicate that RIPC exhibits e protective effect on the Cis-induced AKI animals in a miR-144-dependent manner.

## 4. Discussion

Nephrotoxicity is the principal limitation of the clinical application of cisplatin, inducing renal injuries such as AKI. Even though numerous animal studies on the mechanism of Cis-induced AKI have been performed in rodents, the prevalence of cisplatin-induced AKI inpatients remains extremely high due to a discrepancy between the manifestations of animal models and AKI patients [30]. Therefore, it is significant to identify novel biomarkers and molecular targets of AKI. In this study, we firstly demonstrated that RIPC surgery protects Cis-induced AKI mice from renal injury via activating the PTEN/AKT signaling pathway in miR-144-dependent manner. This provides a possible strategy which is inexpensive and relatively feasible for the prevention of Cis-induced renal injury.

Previously, some groups observed that RIPC exhibits protective effects such as inhibiting renal tubular cell apoptosis and releasing oxidative and inflammatory stress to attenuation of renal dysfunction [25]. The PI3K/PTEN/AKT pathway is a critical mechanism for cells to tackle various cell stresses and control apoptotic program [31]. Also, the PI3K/PTEN/AKT signaling pathway is vital in protecting kidney tubular epithelial cells against apoptosis induced by cisplatin *in vitro* [32]. PTEN, as a negative regulator of PI3K/AKT signaling, was frequently observed being activated in myocardial ischemic injured tissues and reactivation of PI3K/Akt/GSK3*β* signaling by loss function of PTEN attenuated the myocardial ischemic injury [33] In the present study, we also observed that the expression of PTEN (both in mRNA and protein levels) was significantly upregulated in the renal tissues of Cis-induced AKI mice. More importantly, this effect was obviously blocked by RIPC surgery. Recently, Pan et al. also reported RIPC confers renoprotection against septic acute kidney injury by inhibiting the expression of PTEN. Combining these observations, we supposed that PTEN may mediate the renoprotective effects of RIPC surgery. Subsequently, we observed that the elevated phosphorylation level of AKT/GSK3*β* induced by cisplatin administration was blocked by RIPC surgery, which further supports our hypothesis. Then, what is the exact mechanism by which the expression of PTEN is regulated in the process of RIPC surgery?

It was well documented that miRNAs are implicated in many cellular processes such as cell metabolism, division, differentiation, apoptosis, and autophagy [34]. Previously, miR-144 has been demonstrated as an effective upstream regulator of PTEN in distinct contexts, of which the expression also was frequently aberrant in disease animal models, such as I/R-induced cardiomyocyte injury [25]. In the present study, we found that miR-144 was downregulated in the kidney tissues of Cis-induced AKI mice, which was substantially blocked by RIPC surgery. These observations indicated an obvious negative association between the expression of miR-144 and PTEN, suggesting PTEN may be a target of miR-144. Our bioinformatics and experimental results further validated this deduction. Recently, Pan and coworkers also observed that miR-21 directly targets the mRNA of PTEN in the context of the kidney, and the RIPC-induced exosomal miR-21 exerts anti-inflammatory and attenuates sepsis-induced renal injury by activating PTEN/AKT signaling [35]. Considering the redundancy of miRNA, we consider that PTEN may be posttranscriptionally modulated in the synergism of several miRNAs. Noticeably, we observed that the total protein level of PI3K was influenced by cisplatin administration or RIPC surgery in our experiments. Meanwhile, the loss function of miR-144 also caused the alteration of PI3K's expression. We speculated that miR-144 may also target a negative regulator of PI3K.

Finally, we demonstrated that miR-144 is essential for RIPC's neon-protective effects. Our results showed that the loss function of miR-144 by anti-RNA markedly blocked the alleviation of cell apoptosis induced by RIPC surgery. It has been proved that the phosphorylated GSK3*β* inhibits the opening of mitochondrial permeability transition pore (MPTP) and then reduces mitochondrion-dependent apoptosis and necrosis [13, 36]. Indeed, we observed that the phosphorylation of GSK3*β* was upregulated after RIPC surgery, which was dramatically blocked by the loss function of miR-144. Similar results were found in protein level of cleaved caspase-3 and BAX. These findings definitely demonstrated that miR-144 mediates the neon-protective functions of RIPC by inhibiting cell apoptosis.

## 5. Conclusion

We found RIPC surgery can alleviate the renal functional and structural damage of Cis-induced AKI mice. In mechanism, we demonstrated that miR-144 directly targets the mRNA of PTEN and alleviates cisplatin-induced renal cell mitochondrion-dependent apoptosis by reducing the phosphorylated GSK3*β* protein level (Figure 4(g)). Therefore, miR-144 may be a potential biomarker for Cis-induced AKI.

## Data Availability

The data used to support the findings of this study are available from the corresponding authors upon request.
